# Outpatient unicompartmental knee arthroplasty: who is afraid of outpatient surgery?

**DOI:** 10.1007/s00167-017-4440-y

**Published:** 2017-02-22

**Authors:** Alexander Hoorntje, Koen L. M. Koenraadt, Margreet G. Boevé, Rutger C. I. van Geenen

**Affiliations:** Department of Orthopaedic Surgery, Foundation FORCE (Foundation for Orthopaedic Research Care Education), Amphia Hospital, Postbus 90158, 4800 RK Breda, The Netherlands

**Keywords:** Unicompartmental knee arthroplasty, Outpatient surgery, Day care, Fast track, Enhanced recovery, PROMs

## Abstract

**Purpose:**

In recent years, duration of hospitalisation after knee arthroplasty has decreased and fast track and outpatient surgery protocols have been developed. Studies have shown that outpatient surgery is feasible, safe, and cost effective. However, the psychological well-being of patients undergoing outpatient surgery has never been described before. The purpose of this study was to investigate how patients experience outpatient surgery for unicompartmental knee arthroplasty (UKA), examining levels of anxiety and depression, satisfaction, and pain. It was hypothesized that the same-day discharge following UKA would not result in higher levels of anxiety and depression, compared to the standard fast-track surgery.

**Methods:**

This case-controlled study included 20 patients undergoing UKA in an outpatient surgery setting and 20 patients undergoing the standard fast-track procedure. The Hospital Anxiety and Depression Scale (HADS, 0–42, lower is better) and numeric rating scales (NRS, 0–10) for pain and satisfaction were collected preoperatively, on the day of surgery, on the first, second, and seventh postoperative days and after 6 and 12 weeks. The Oxford Knee Score (OKS), the KOOS, EuroQoL-5D, and Net Promoter Score (NPS) were collected preoperatively and 3 months postoperatively.

**Results:**

90% of patients in the outpatient surgery group were discharged on the day of surgery. At the first postoperative day, the median HADS score was significantly lower in the outpatient surgery group compared to the fast-track group (3 vs. 8, *p* = 0.02), the median NRS satisfaction score was significantly higher in the outpatient surgery group (8 vs. 5, *p* = 0.03), and no differences existed between both groups for the NRS pain scores. At 3 month follow-up, no significant differences in improvement scores existed between both groups for the HADS, the NRS scores, and for the OKS, KOOS, EuroQoL-5D, and NPS.

**Conclusion:**

The results of this study emphasize the feasibility of an outpatient surgery pathway in carefully selected UKA patients. The outpatient surgery pathway is safe, and clinical outcome, including levels of anxiety and depression, satisfaction, and pain, was similar in outpatient surgery patients compared to the standard fast-track patients.

**Level of evidence:**

Case-control study, Level III.

## Introduction

For decades, unicompartmental knee arthroplasty (UKA) was considered a surgical procedure requiring prolonged hospitalisation periods, but in recent years, the shortening of hospitalisation after UKA has gained considerable interest. Already, the average length of stay has markedly decreased with the implementation and optimisation of postoperative fast-track pathways [2, 17]. Fast-track UKA allows for safe, efficient care with fewer perioperative complications and early discharge, which in turn leads to higher patient satisfaction [1, 4, 14, 17]. The average reported length of stay in fast-track programs for UKA patients has already decreased to 1 day, with good results [23].

Therefore, the introduction of outpatient surgery seemed like the logical next step in attempting to further improve clinical outcome and shortening length of stay in UKA. Several authors have described the use of an outpatient surgery pathway in UKA and so far results have been very promising [1, 7, 9, 11, 21]. Discharge on the day of surgery was possible in almost all cases, varying from 85 to 100% of cases. Furthermore, incidence of adverse events, complications, and readmissions was low and rates were comparable to UKA patients operated on in a fast-track pathway [2, 7, 11, 21].

While the above-mentioned studies primarily focused on clinical outcome in terms of safety (adverse events, complications), practical challenges, and feasibility of outpatient surgery pathways for UKA, patients’ levels of anxiety and depression when undergoing UKA in an outpatient setting have not been described before. Interestingly, the previous research showed that, in 135 patients undergoing different types of elective procedures, outpatient surgery patients experienced significantly higher perioperative levels of anxiety and depression compared to fast-track patients [26]. The presence of psychological symptoms, such as anxiety and depression, may negatively influence surgical outcome following KA [12, 14, 18]. Thus, it is very important to ascertain that an outpatient surgery pathway for UKA is also safe in terms of the patients’ psychological well-being. However, none of the previous studies on outpatient surgery for UKA have addressed the effect of an outpatient protocol on the patients’ psychological well-being.

Therefore, the effect of an outpatient surgery pathway for UKA was investigated, comparing the levels of anxiety and depression, pain, and satisfaction that patients experienced perioperatively, compared to the standard fast-track treatment. Based on the excellent results from the previous outpatient surgery studies in UKA patients, it was hypothesized that the same-day discharge following UKA in carefully selected patients would not result in higher levels of anxiety or depression, more pain or lower satisfaction, compared to a fast-track pathway.

## Materials and methods

### Study design and patient population

**Fig. 1:**
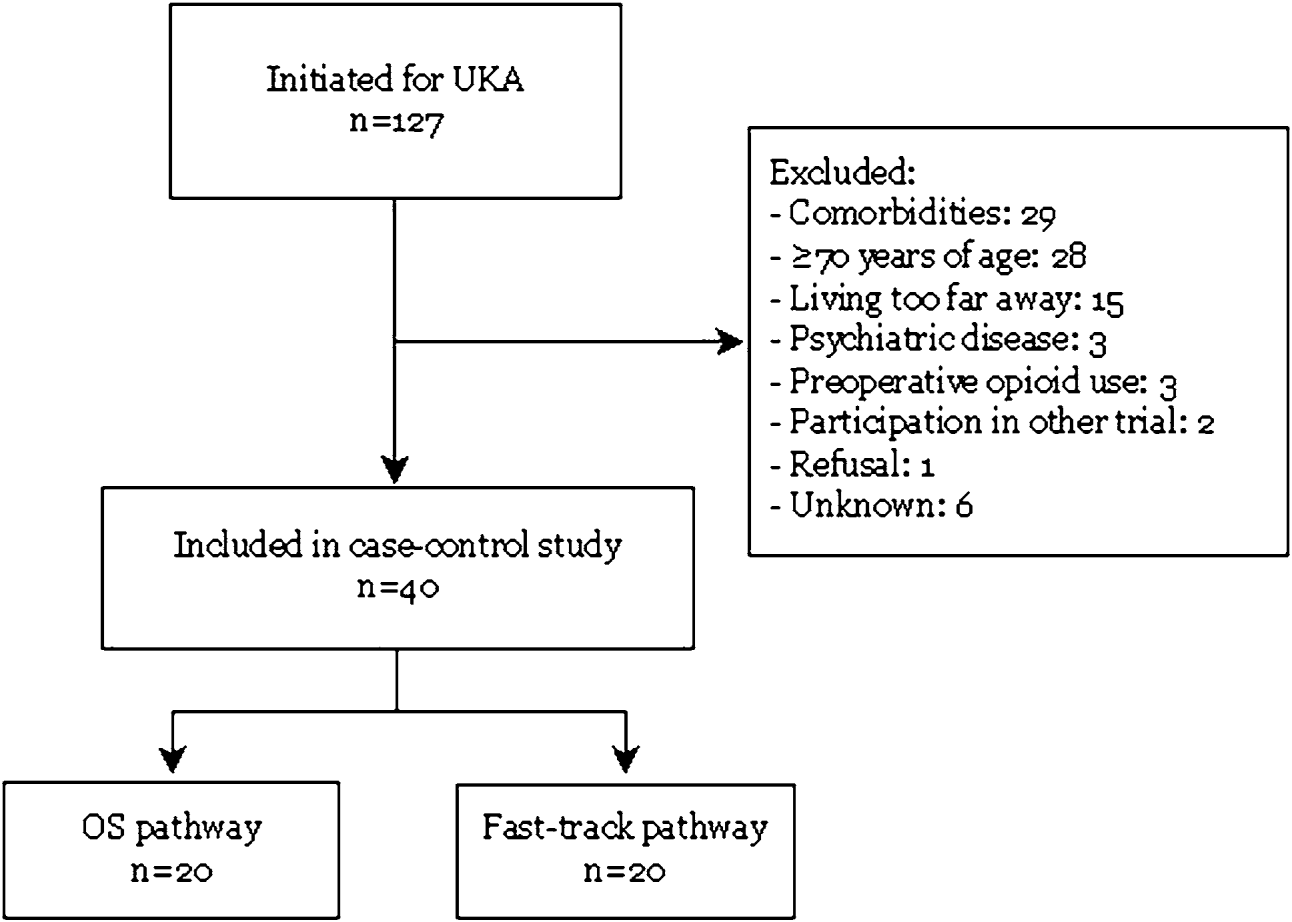
Flowchart for the screening and enrolment process

In this case-control study, 20 patients undergoing UKA in an outpatient surgery setting and 20 patients undergoing UKA in a standard fast-track setting between June 2015 and June 2016 were compared. Baseline characteristics are shown in Table 2. The study was performed in compliance with the Helsinki Declaration of 1975, as revised in 2000. Eligible patients were <70 years of age, ASA 1–2, and motivated to participate in the outpatient surgery program. A personal coach (relative) had to be available during the first 24 h after discharge to assist the patient at home in the first postoperative phase. Figure 1 presents the flowchart for the screening and enrolment process. Patients with a BMI higher than 35 kg/m^2^ or with a history of diabetes, recent myocardial infarction, congestive heart failure, stroke, thromboembolic events, respiratory disease, or opiate use were excluded. In addition, patients with a history of mental illness (depression and anxiety disorders) were excluded. Finally, patients living too far away from the hospital for the home visit by the hospital physiotherapists were excluded.

### Standard fast-track protocol

Table 2 shows the differences between the fast-track protocol and the outpatient surgery protocol. Approximately 1 h before surgery, all patients received paracetamol (1 g), Meloxicam (15 mg), Pantoprazole (40 mg), and Gabapentin (300 mg). All patients received the uncemented Oxford phase III prosthesis (Biomet, Bridgend, UK). Surgery was performed using patient-specific guides (Signature, Biomet, Warsaw INC) in four fast-track patients and in three outpatient surgery patients. The Microplasty instrumentation (Biomet, Bridgend, UK) was used in 16 fast-track patients and 17 outpatient surgery patients. Cefazolin was used as perioperative prophylactic antibiotics for 24 h. Patients were either operated under spinal or general anaesthesia (Table 1). All patients received a preoperative dose of tranexamic acid. Local infiltration analgesia (LIA) was administered intraoperatively. To prevent postoperative nausea, perioperative dexamethasone (8 g) was administered. At wound closure, another dose of tranexamic acid (1 g) was administered. No drains or urinary catheters were used. To reduce swelling and improve LIA effect, a compression bandage was used [6] (Table 1). Postoperatively, patients with a urinary retention over 500 mL were catheterised. For postoperative pain management, meloxicam (15 mg once daily) and paracetamol (1000 mg four times daily) were prescribed. In addition, in the fast-track protocol, oxycodone (10 mg) was administered 4–6 times daily on the first postoperative day. The hospital physiotherapist visited the patient two times on the day of surgery (two and 4 h postoperatively), and normal walking and walking stairs were practiced on the first postoperative day. Thromboprophylaxis was prescribed for 6 weeks and conforms national guidelines. All patients were seen at the outpatient clinic after 14 days and after 6–8 weeks.

**Table 1:** Baseline characteristics

Variable	Outpatient surgery (*n* = 18)	Control (*n* = 18)	*P* value^a^
Age at surgery, years	62.2 ± 5.5	63.8 ± 7.5	n.s.
Gender, male	10 (56%)	7 (37%)	n.s.
BMI, kg/m^2^	27.8 ± 3.7	30.5 ± 7.0	n.s.
ASA			n.s.^b^
1	7 (39%)	6 (32%)	
2	11 (61%)	10 (53%)	
3	0	3 (15%)	
4	0	0	
OR time, min	62.6 ± 13.1	60.5 ± 20.8	n.s.
Anaesthesia			n.s.
General	10	12	
Spinal	10	8	
Surgical technique			n.s.
Signature	3	4	
Microplasty	17	16	
LOS (days)	0	1.3 (1–4)	–

^a^Comparison with Chi-square test, Fisher’s exact test, or Independent samples *T* test

^b^Fisher–Freeman–Halton test

**Table 2:** Differences between the outpatient surgery pathway and the fast-track pathway

	Fast-track pathway	Outpatient surgery pathway
Preoperative		
Patient education	Group education	Individual education
Perioperative		
Antibiotics	IV (preoperatively, 8 and 16 h postoperatively)	IV (preoperatively and 8 h postoperatively)
Postoperative		
Compression bandage	16–24 h (removed on ward)	24 h (removed by physiotherapist)
Physiotherapy by hospital physiotherapist	After 2, 4 h and on postoperative day 1	After 2, 4, 6 h At home on day 1
Opioid use	Oxycodone 5–10 mg (4–6 times daily)	–
Discharge criteria*	–	Independent transfers and independent walking

*Standard discharge criteria applied to all patients: no or limited wound drainage, acceptable pain level, no medical indication for prolonged hospital stay, patient feels confident going home

### Outpatient surgery protocol

In contrast to the fast-track pathway, patients in the outpatient surgery pathway had a personal educational meeting with a nurse practitioner to avoid confusion in the standard group educational meetings (Table 1). Preferably, the patients’ coach was also present at this meeting. Instructions on the outpatient surgery process, physiotherapy, and the rehabilitation protocol were provided, and patients’ questions and expectations were addressed. No preoperative exercise training or rehabilitation was provided, but patients were advised to contact a physiotherapist to discuss postoperative arrangements.

All surgeries in the outpatient surgery group were performed by one experienced knee surgeon (RvG). All outpatient surgery patients were operated on in the morning to allow for completion of the entire postoperative rehabilitation protocol in the hospital. In contrast to the fast-track protocol, an opioid-sparing multimodal pain protocol was used. Only in case of breakthrough pain, oxycodone 5 mg (max. 4 times daily) was administered as rescue pain medication. Two hours postoperatively, patients were seen by a physiotherapist and knee flexion and extension was practiced. After 4 h, patients were mobilised with help of the physiotherapist. Mobilisation included transfers from bed to chair, standing, and walking with the use of an assistive device (walker, crutches). After 6 h, walking was practiced again, including walking stairs if this was required. The following discharge criteria were used: no or limited wound drainage, acceptable pain level, no medical indication for prolonged hospital stay, independent transfers in and out of bed, independent walking, and, if necessary, walking stairs. Patients were allowed to go home under the supervision of their coach. The treating surgeon visited each patient before discharge. At the first postoperative day, a physiotherapist from the hospital visited the patient at home to remove the compression bandage, to explain and practice rehabilitation exercises, and to evaluate the day of surgery. After this visit, patients continued their rehabilitation with their own physiotherapist. At days 2 and 7, a nurse from the orthopaedic ward called the patient to check if there were any problems or complications.

### Outcome measures

Outcome in terms of adverse events, opiate use, and complications was carefully monitored. PROMs were collected preoperatively, at discharge, at home on the evening of the day of surgery, at three moments on the first postoperative day (morning, afternoon, and evening), and at postoperative days 2 and 7, at 6 weeks and at 3 months postoperatively. The Hospital Anxiety and Depression Scale (HADS, 0–42, lower is better) was used to assess patients’ levels of anxiety and depression [27]. The HADS is a 14-item questionnaire with seven items (0–21 points) addressing patients’ anxiety level and seven items addressing depression (0–21 points). It is the preferred measure of anxiety and depression for non-psychiatric hospital patients. Cut-off points for the presence of anxiety disorders or depression have been investigated [3]. A score of ≥ 8/21 points indicates the presence of an anxiety disorder and/or depression. Numeric rating scales (NRS, 0–10) were used to assess patient satisfaction, pain at rest, and pain after mobilisation. In addition, the Dutch validated versions of the Oxford Knee Score (OKS, 12–60, lower is better), Knee Injury and Osteoarthritis Outcome Score (KOOS, 5 subscales with scores 0–100, higher is better), and the EuroQol-5D VAS health score (EQ-5D; 0–100, higher is better) [5, 8, 24] are routinely collected preoperatively and 3 months postoperatively. Finally, the Net Promoter Score (NPS, 0–10), which evaluates how likely patients would recommend the operation to a relative or close friend, was collected [13].

### Statistical analysis

All statistical analyses were performed with SPSS for Windows (Version 24.0. Armonk, NY: IBM Corp). Since the fast-track pathway and outpatient surgery pathway mainly differ on the first postoperative day, differences in HADS and NRS scores were compared with Mann–Whitney *U* tests at that timepoint. Mean HADS scores on the first postoperative day were 4.1 for the OS group and 9.3 for the fast-track group, with an SD of 5.2. For an expected difference of 5.2 points on the HADS at day 1 with an SD of 5.2, with a two-sided significance of 0.05 and a power of 0.8, a total of 20 subjects in each group would be required (nQuery Advisor® version 7.0). Differences from baseline to 3-month follow-up within each separate group were analysed with Wilcoxon signed-rank test. For all outcome parameters, differences between the outpatient surgery group and fast-track group, from baseline to 3-month follow-up, were analysed.

## Results

Of the 20 patients included in the outpatient surgery group, 18 patients (90%) could go home on the day of surgery. In one case, the required prosthesis was not available on the OR at the scheduled time of surgery, and thus, the operation was delayed. Therefore, the rehabilitation protocol in the hospital could not be completed and the patient had to stay for one night. In one case, anaesthesiologists disagreed on the ASA classification of the patient, who had a history of cardiac events. Therefore, it was decided on the OR that the patient had to stay for one night. Thus, 18 patients were included for analysis in the outpatient surgery group. Postoperatively, one patient in the outpatient surgery group visited the ER on the first postoperative day due to wound leakage. Two extra sutures were placed, and the patient could return home. In the fast-track group, 18 patients completed the questionnaires sufficiently and two patients were excluded due to insufficient data. The average length of stay in the control group was 1.3 days (range 1–4).

### Patient-reported outcome measures

**Fig. 2:**
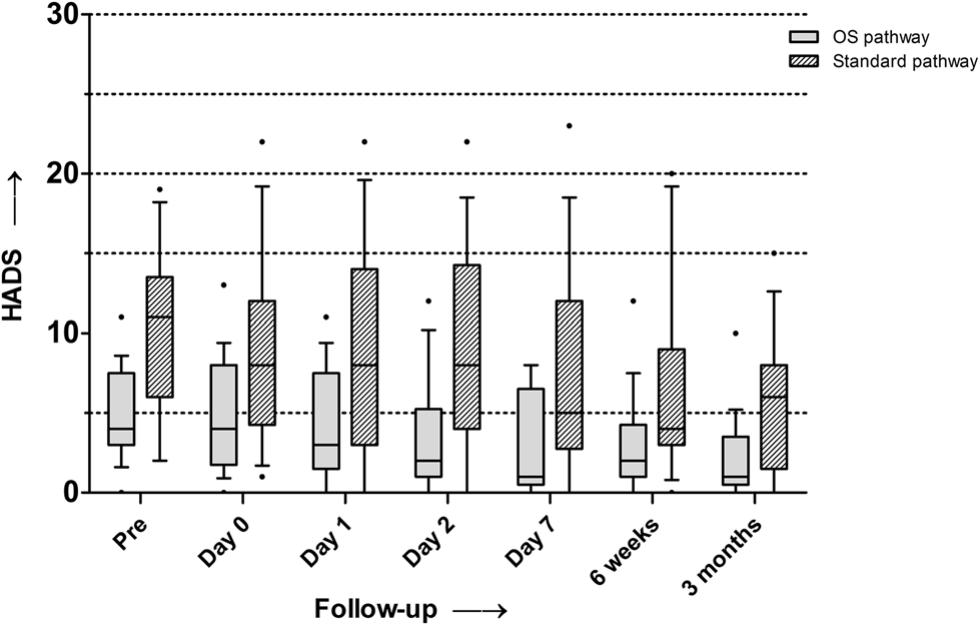
Median HADS scores for both groups (*boxes* indicate the first and third percentiles; *whiskers* indicate 10–90‰; *dots* indicates outliers)

Figure 2 shows boxplots of the median HADS scores for both groups at all timepoints. Both groups showed a decrease, i.e., improvement, in HADS scores over time. The median HADS score appeared to be lower in the outpatient surgery group at all timepoints (Fig. 2). At day 1, the median HADS score was significantly lower (*p* = 0.02) in the outpatient surgery group (3.0, range 0–11) compared to the fast-track group (8.0, range 0–22). At the final follow-up, the median HADS score in the outpatient surgery group decreased from 4.0 to 1.0 (range 0–10, *p* < 0.01). In the fast-track group, the median HADS score decreased from 11.0 to 6.0 (range 0–15, *p* < 0.01). No significant difference was found between the groups in improvement of the HADS at final follow-up.

**Fig. 3:**
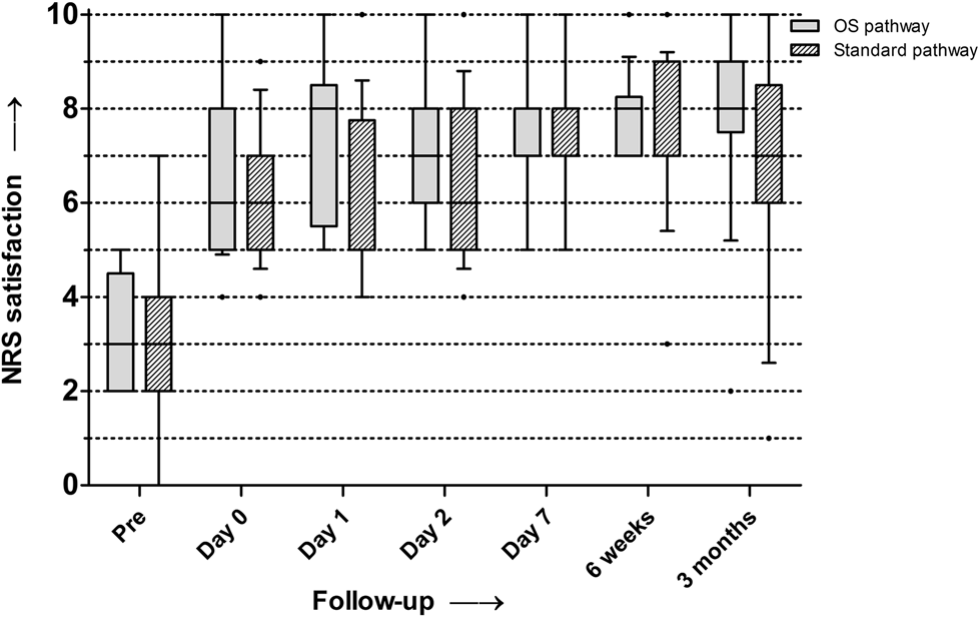
Median NRS satisfaction scores for both groups (*boxes* indicate the first and third percentiles; *whiskers* indicate 10–90‰; *dots* indicate outliers)

Figure 3 shows boxplots of the median NRS satisfaction scores. Higher satisfaction scores over time were observed for both groups independently (Fig. 2). At day 1, the median NRS satisfaction score was significantly higher (*p* = 0.03) in the outpatient surgery group (8, range 5–10) compared to the fast-track group (5, range 4–10). At the final follow-up, the median NRS satisfaction score in the outpatient surgery group improved from 3 to 8 (range 2–10, *p* < 0.001) and in the fast-track group from 3 to 7 (range 1–10, *p* < 0.01). No significant difference was found between the groups in improvement of the NRS satisfaction score at the final follow-up.

**Fig. 4:**
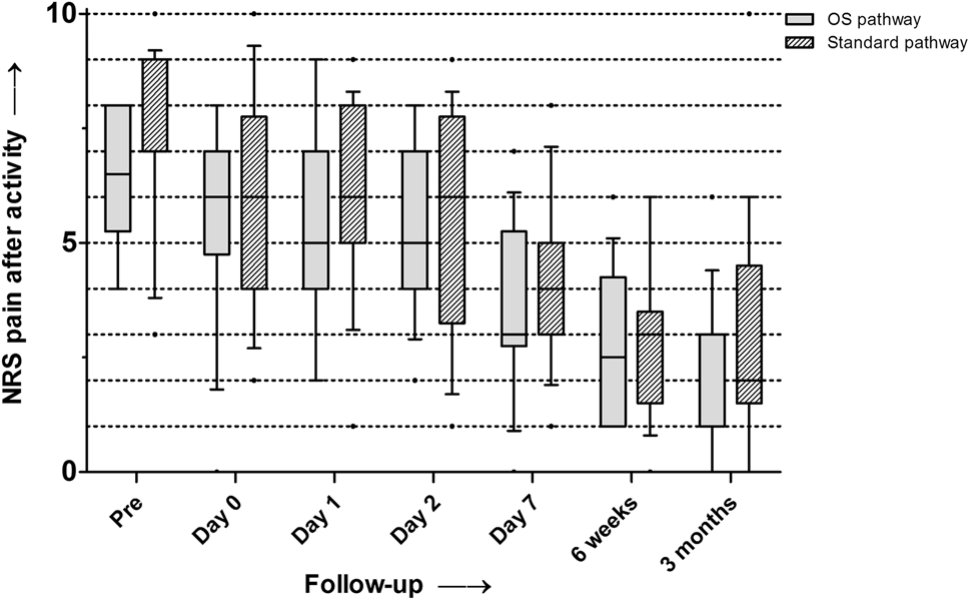
Median NRS pain after activity scores for both groups (*boxes* indicate the first and third percentiles, and *whiskers* indicate the range)

The NRS pain after activity scores decreased for both groups independently (Fig. 4). At day 1, the median NRS pain after activity score was not significantly different between both groups. At the final follow-up, the median NRS pain after activity score in the outpatient surgery group decreased from 8 to 3 (range 0–6, *p* < 0.01) and in the fast-track group from 7 to 3 (range 0–10, *p* < 0.01). No significant difference was found between the groups in improvement of the NRS pain after activity score at the final follow-up. The NRS pain in rest scores showed the same pattern as the NRS pain after activity scores at all timepoints.

Table 3 presents the improvement scores for the KOOS, OKS, EQ-5D VAS scores, and the NPS. Improvement for the KOOS symptoms subscale was greater in the fast-track group, but the absolute score did not differ significantly (80 vs. 84, n.s.).

**Table 3:** Improvement scores for the KOOS, OKS, and EQ-5D in both groups

PROM	Outpatient surgery group mean (SD)	Control group mean (SD)	*P* value^a^
ΔKOOS pain	28.0 (17.0)	35.6 (25.5)	n.s
ΔKOOS symptoms	19.3 (19.1)	33.2 (20.7)	0.04*
ΔKOOS ADL	23.2 (23.0)	37.8 (21.4)	n.s
ΔKOOS sport	28.9 (30.0)	35.8 (29.9)	n.s
ΔKOOS QoL	33.8 (17.6)	39.9 (23.0)	n.s
ΔOKS	12.0 (5.7)	16.1 (10.7)	n.s
ΔEQ-5D VAS	15.0 (24.4)	16.2 (21.5)	n.s.^b^
NPS	47	47	–

*Significance is assumed at *p* < 0.05

^a^Independent samples *t* test

^b^Mann–Whitney *U* test

## Discussion

The most important finding of the present study was that UKA could be successfully performed in an outpatient setting with regard to the patients’ psychological well-being. In carefully selected patients, outpatient surgery did not compromise outcome in terms of levels of anxiety and depression, satisfaction and pain scores. There were no medical complications that prohibited patients from going home in the outpatient surgery group, and 18 (90%) patients could go home on the day of surgery.

The present study is only the second study on outpatient surgery to include a control group, allowing for a comparison between fast-track surgery, which is currently seen as the golden standard and outpatient surgery [14]. More importantly, this study is the first to describe the presence of symptoms of anxiety and depression, by means of the HADS, in UKA patients. The previous studies have shown that the presence of psychological symptoms in KA patients resulted in increased LOS, readmissions and morbidity, and worse patient-reported outcomes [12, 18]. Duivenvoorden et al. found that HADS scores decreased over time in TKA patients, but patients with high preoperative HADS scores had worse PROMs at 3 and 12 months [10]. In the present study, HADS scores showed a similar decrease, i.e., less anxiety and depression, in both groups over time. Remarkably, significantly lower HADS scores were found in the outpatient surgery group compared to the fast-track group. The difference in HADS scores may be partly explained by the individual education meeting for outpatient surgery patients [16, 22]. During this meeting, an extensive explanation of the outpatient surgery procedure was given, and therefore, the patient knew exactly what to expect. In addition, after the operation, the hospital physiotherapist visited the patient more often. This additional personal attention may have led to outpatient surgery patients feeling more confident and less anxious. Finally, it is possible that patients in the outpatient surgery group were already less anxious preoperatively, since less anxious patients presumably would be more willing to undergo outpatient surgery. Nevertheless, the most important conclusion of the above-mentioned findings is that an outpatient surgery pathway for UKA does not appear to compromise patients’ psychological well-being.

In addition to the HADS sores, the present study is the first to describe satisfaction scores at several timepoints in the direct postoperative phase. Kolisek et al. found that satisfaction scores in UKA patients undergoing outpatient surgery or fast-track surgery did not differ at 24 months [19]. However, it seems unlikely that an additional effect of outpatient surgery on satisfaction would still be present after 24 months. The present study showed that satisfaction scores in the direct postoperative phase, up until 3 months follow-up, were equal or even better in outpatient surgery patients, compared to fast-track patients. Furthermore, no significant differences in pain scores existed, indicating that pain scores were not influenced by the early discharge to the home environment for outpatient surgery patients. This is in line with reported pain scores in the only other case-control study by Kort et al. showing that outpatient surgery and fast-track surgery result in similar pain scores [21]. In contrast, high pain intensity was the main factor for an overnight stay in the study by Kort et al., while none of our patients experienced pain that prevented them from going home on the day of surgery. Finally, the improvement for the KOOS symptoms’ subscale was significantly higher in the fast-track pathway compared to the outpatient surgery pathway. However, the KOOS and OKS scores were already higher preoperatively in the outpatient surgery group and no significant differences existed between both groups in overall KOOS and OKS scores at final follow-up. In conclusion, outpatient surgery patients were very satisfied and performed at least as good as fast-track surgery patients.

Concerning the general applicability of the above-mentioned studies, it is important to note that the authors had already implemented fast-track surgery as the standard pathway for hip and knee arthroplasty in their institutions. Accordingly, the hospitals’ staff had experience with local infiltration analgesia, multimodal opioid-sparing anaesthetic regimens, mobilising patients on the day of surgery, and checking the standard discharge criteria twice a day to prevent unnecessary hospitalisation. In addition, different authors stress the importance of a dedicated team of surgeons, anaesthesiologists, physiotherapists, and nursing staff when implementing outpatient pathways. Implementation of an outpatient pathway, as experienced by us, Berger et al. and Kort et al., required an extensive change in mind-set for both the patients and the multidisciplinary team. It is, therefore, recommended to gradually reduce LOS from ≥2 days to next-day discharge first. Subsequently, only when staff and patients are comfortable with the next-day discharge, the same-day discharge can be carefully attempted [1].

Finally, patient selection in KA is an important topic in the recent literature, since proper selection is considered essential in assuring patient safety and preventing negative outcome and dissatisfaction [20, 25]. Two recent studies have pointed out that well-conducted patient selection in outpatient surgery is very important to assure a safe procedure. Based on a literature review [20] and a retrospective review of patient characteristics associated with same-day discharge [25], the authors stated that exclusion criteria for outpatient joint arthroplasty should include: high ASA classification (>II), bleeding disorders, poorly controlled, and/or severe cardiac (e.g., congestive heart failure and arrhythmia) or pulmonary comorbidities (e.g., embolism and respiratory failure), uncontrolled DM (type I or II), chronic opioid consumption, functional neurologic impairments, dependent functional status, chronic/end-stage renal disease, and/or reduced preoperative cognitive capacity. However, both authors observed a void in literature concerning proper selection criteria for outpatient knee arthroplasty. The additional risk of outpatient surgery compared to a fast-track pathway, which is considered standard care, may not justify much stricter inclusion criteria. Recently, Jorgensen et al. showed that the incidence of early (<7 days) thromboembolic events, the main life threatening early complication postoperatively, was seen in 11/13.775 unselected patients (0.23%) undergoing knee or hip replacement [15]. Patients were discharged after a mean LOS of 2 days. Out of 43 thromboembolic events, 11 events occurred in the first two postoperative days and only two events occurred after discharge. This study illustrates that, if a patient is considered eligible for standard fast-track KA surgery, the additional risk of an outpatient surgery pathway appears to be negligible.

A limitation of the present study is the fact that patients were not randomized. In accordance with several other studies describing KA in an outpatient setting, a case-control study was performed [19, 21]. Strict inclusion and exclusion criteria were used to ensure a safe introduction of our novel outpatient surgery pathway [20]. It cannot be ruled out that patients in the outpatient surgery pathway were healthier as a group at the time of surgery. Therefore, randomized controlled trials should be conducted to eliminate possible confounders, such as the allocation of patients with less severe symptoms to the outpatient surgery pathway.

## Conclusion

In conclusion, the findings of this study suggest that outpatient surgery for UKA is a safe and attractive treatment option in selected, motivated patients. This is a clinically relevant finding that will aid the orthopaedic surgeon in the decision to implement outpatient surgery for UKA. The patients’ psychological well-being appears to influence outcome and should be taken into account when selecting patients for outpatient surgery. Future studies, including case series with larger numbers of patients and, most importantly, randomized controlled trials, are necessary to endorse findings of the present case–control study.
